# Belantamab Mafodotin to Treat Multiple Myeloma: A Comprehensive Review of Disease, Drug Efficacy and Side Effects

**DOI:** 10.3390/curroncol28010063

**Published:** 2021-01-21

**Authors:** Grace Lassiter, Cole Bergeron, Ryan Guedry, Julia Cucarola, Adam M. Kaye, Elyse M. Cornett, Alan D. Kaye, Giustino Varrassi, Omar Viswanath, Ivan Urits

**Affiliations:** 1 School of Medicine, Georgetown University, Washington, DC 20007, USA; 2 School of Medicine, Louisiana State University Shreveport, Shreveport, LA 71103, USA; cberg7@lsuhsc.edu (C.B.); rgued1@lsuhsc.edu (R.G.); jcucar@lsuhsc.edu (J.C.); 3 Department of Pharmacy Practice, Thomas J. Long School of Pharmacy and Health Sciences, University of the Pacific, Stockton, CA 95211, USA; akaye@pacific.edu; 4 Department of Anesthesiology, Louisiana State University Shreveport, Shreveport, LA 71103, USA; ecorne@lsuhsc.edu (E.M.C.); akaye@lsuhsc.edu (A.D.K.); viswanoy@gmail.com (O.V.); ivanurits@gmail.com (I.U.); 5 Paolo Procacci Foundation, Via Tacito 7, 00193 Roma, Italy; giuvarr@tin.it; 6 College of Medicine-Phoenix, University of Arizona, Phoenix, AZ 85724, USA; 7 Department of Anesthesiology, School of Medicine, Creighton University, Omaha, NE 68124, USA; 8 Valley Anesthesiology and Pain Consultants—Envision Physician Services, Phoenix, AZ 85004, USA; 9 Southcoast Health, Southcoast Physicians Group Pain Medicine, Wareham, MA 02571, USA

**Keywords:** belantamab mafodotin, multiple myeloma, anti-B cell maturation antigen, antibody drug conjugate, chronic pain

## Abstract

Multiple myeloma (MM) is a hematologic malignancy characterized by excessive clonal proliferation of plasma cells. The treatment of multiple myeloma presents a variety of unique challenges due to the complex molecular pathophysiology and incurable status of the disease at this time. Given that MM is the second most common blood cancer with a characteristic and unavoidable relapse/refractory state during the course of the disease, the development of new therapeutic modalities is crucial. Belantamab mafodotin (belamaf, GSK2857916) is a first-in-class therapeutic, indicated for patients who have previously attempted four other treatments, including an anti-CD38 monoclonal antibody, a proteosome inhibitor, and an immunomodulatory agent. In November 2017, the FDA designated belamaf as a breakthrough therapy for heavily pretreated patients with relapsed/refractory multiple myeloma. In August 2020, the FDA granted accelerated approval as a monotherapy for relapsed or treatment-refractory multiple myeloma. The drug was also approved in the EU for this indication in late August 2020. Of note, belamaf is associated with the following adverse events: decreased platelets, corneal disease, decreased or blurred vision, anemia, infusion-related reactions, pyrexia, and fetal risk, among others. Further studies are necessary to evaluate efficacy in comparison to other standard treatment modalities and as future drugs in this class are developed.

## 1. Introduction

Multiple Myeloma (MM) is a hematologic malignancy characterized by the clonal proliferation of plasma cells. This disease overproduces terminally differentiated B-cells and immunoglobulins, which crowd the bone marrow and result in end-organ damage and immune suppression. Consequently, as levels of monoclonal paraproteins accumulate, patients suffer from anemia, infections due to immune cell depletion, bone pain, increased calcium levels, and renal failure [1,2,3]. Multiple myeloma develops stepwise from premalignant conditions, known as the monoclonal gammopathy of undetermined significance (MGUS) and smoldering multiple myeloma (SMM). These conditions are defined by detectable paraproteins in the blood and urine, do not cause end-organ damage, and, therefore, do not require treatment [2]. However, MGUS and SMM have the potential to develop into MM once paraproteins accumulate to levels high enough to cause damage to the bone marrow, kidney, and other organs. With the presence of the clonal proliferation of plasma cells and evidence of end-organ damage, the diagnosis of MM can be made.

The global incidence of multiple myeloma has markedly increased in recent years, accounting for about 1.8% of all cancer cases [4,5]. This disease remains incurable for many patients and requires novel drugs to combat the associated high mortality rates. Five-year survival rates for MM have dramatically improved over the past decade with the advancement of treatment options such as stem cell transplants, proteasome inhibitors, histone deacetylase inhibitors, monoclonal antibodies, and immunomodulators [4,6]. However, the treatment of multiple myeloma presents a variety of unique challenges due to the complex molecular pathophysiology, intra-clonal heterogeneity, and the incurable status of the disease at this time. While the majority of patients respond to initial treatment, most will relapse, and response to further treatment declines with subsequent relapses [1].

There is no current cure for multiple myeloma despite the development of targeted biologic agents and immunotherapy in the past decade. Relapse and refractory disease remain considerable challenges in the pharmacologic management of the disease. The first drug in its class, belantamab mafodotin-blmf (brand name Blenrep) is a novel therapeutic agent recently approved by the FDA to treat relapsed or treatment-refractory multiple myeloma. Belamaf is an immunoconjugate anti-B-cell maturation antigen with high specificity for multiple myeloma cells **[7]**. For those with limited treatment options, belamaf has the potential to dramatically change patient outcomes. The recent FDA approval of this drug emphasizes the continued need for innovative medications with high safety profiles, particularly those that improve quality of life and mitigate multiple myeloma disease progression.

## 2. Multiple Myeloma Epidemiology and Risk Factors

Multiple myeloma (MM) is a rare cancer that accounts for 1.8% of all new cancer cases in the United States [2]. After lymphoma, MM is the second most common malignancy of the blood cell lineage [8]. According to the National Cancer Institute, there will be an estimated 32,270 new cases and 12,830 deaths (2.1% of all cancers) in 2020 in the United States [9]. All cases of MM begin as the benign condition called monoclonal gammopathy of undetermined significance (MGUS). MGUS can progress to smoldering multiple myeloma (SMM), which has the potential to progress to MM. These conditions are characterized by an increase in the amount of monoclonal immunoglobulin produced, but which are asymptomatic and do not cause end-organ damage [2]. The risk of progression from MGUS and SMM to the malignant form of MM is 1% and 10% per year, respectively [2]. In rare cases, MM has the ability to progress further into Plasma Cell Leukemia (PCL), where plasma cells begin to proliferate outside the bone marrow and accumulate in the bloodstream.

Older age, male sex, and African American race are the leading risk factors for the development of MM. Data from the NIH show that the age-adjusted rates of new cases per 100,000 were higher in males (8.8%) than females (5.7%), and highest in African Americans for both sexes [5]. The incidence of MM is correlated to age, with a median age of 69 and the majority of patients diagnosed between 65–74 years. Multiple myeloma is rare in patients younger than 30 years [5]. Other factors associated with increased risk include alcohol consumption, obesity, radiation exposure, and insecticides [2]. New effective therapy to treat multiple myeloma has increased 5-year survival rates from 25% in the years 1975–1977 and 27% in 1987–1989 to 49% in the period 2005–2011 and 53.9% in the period 2010–2016 [5,10]. The increase in survival rates is attributed to the approval of the autologous stem cell transplant (ASCT), bortezomib, and thalidomide/lenalidomide [6].

## 3. Pathophysiology

Multiple myeloma begins as a clonal proliferation of post germinal center B-cells that produce large amounts of monoclonal immunoglobulin and light chain protein. Primary genetic events trigger disease progression, with most cases of MM being due to chromosomal translocations involving the immunoglobulin-heavy chain (IGH) genes (which interfere with antibody class switching) and aneuploidy [11]. Other genetic events involved in the pathogenesis of MM include chromosome 13 deletion, activating RAS and BRAF mutations, the dysregulation of MYC, mutations in the NF-kB pathway, chromosome 17p loss with abnormalities in p53, a gain of chromosome 1q and a loss of 1p [12,13]. Of note, myeloma cells interact with the immune and bone marrow microenvironment, leading to the uncoupling of bone remodeling. This process may be partially explained by the activation of osteoclasts (RANKL/RANK interactions, increased MIP-1α, decreased osteoprotegerin) and the suppression of osteoblast formation and differentiation (dysregulation of factors Dkk1, sFRP-2, IL-3, Runx2 and TGF-β) [12]. Furthermore, multiple myeloma exhibits clonal heterogeneity and clonal evolution, which impacts clinical presentation and drug sensitivity. The initial disease may be characterized by a predominance of one clone that responds well to treatment. Yet another inciting event may allow for a new clone with novel genomic abnormalities to emerge, resulting in disease relapse. Some patients have been shown to have up to seven subclones [11,14].

Almost all cases of MM begin as the premalignant conditions Monoclonal Gammopathy of Undetermined Significance (MGUS) and Smoldering Multiple Myeloma (SMM). MGUS and SMM are asymptomatic conditions that may progress to multiple myeloma if levels of monoclonal protein rise and genetic alterations in plasma cells accumulate [3]. MGUS is clinically distinguished from MM as having a serum monoclonal Ig of less than 3 g/dL, a bone marrow plasma cell content of less than 10%, no evidence of end-organ damage, and a 1% annual risk of progression to MM. SMM is defined as having a serum monoclonal Ig of greater than 3 g/dL, a bone marrow plasma cell content of greater than 10%, and no detectable end-organ damage, and a 10% annual risk of progression to MM [12]. By acquiring any combination of the oncogenic events mentioned above, MGUS and SMM are able to progress to MM, the malignant form of the disease.

## 4. Presentation

According to current IMWG guidelines, the diagnostic criteria for symptomatic multiple myeloma includes one or more myeloma-defining events (MDE) in addition to either clonal bone marrow plasma cells ≥10% or biopsy-confirmed bony or extramedullary plasmacytoma [15]. MDE include one or more CRAB criteria (hypercalcemia, renal failure, anemia, lytic bone lesions) or one or more biomarkers of malignancy. Laboratory analysis is key to diagnosis, as patients often present with vague symptoms, such as nausea, vomiting, malaise, weakness, recurrent infections, or weight loss [3,16]. In a majority of cases, laboratory tests show anemia, hypercalcemia, and/or proteinuria [3].

As plasma cells begin to proliferate, the bone marrow becomes crowded, and the ability of other cell lines to produce mature cells can lead to anemia, thrombocytopenia, and neutropenia [1]. Anemia is present in almost all patients during the disease course and can present with fatigue, dyspnea, or angina [16,17]. A blood smear will show normochromic and normocytic anemia (mild macrocytosis may be seen) with the possible rouleaux formation of red blood cells [17]. Hypercalcemia occurs less frequently at the time of diagnosis. When symptomatic hypercalcemia arises, patients may present with confusion, muscle weakness, constipation, anorexia, polyuria, and polydipsia. If levels rise to dramatically high levels, severe complications, such as cardiac arrythmias or coma, may occur [17,18].

As the level of the immunoglobulin light chain rises, the protein may deposit in the distal and collecting renal tubules, causing renal impairment with a resulting rise in serum creatine levels (≥2 mg/dL). Hypercalcemia and the use of NSAIDs can also contribute to renal impairment [17,19]. Patients may experience bone pain, commonly back pain. Lytic bone lesions, found in up to 80% of cases, contribute to hypercalcemia due to increased osteoclast activity and bone resorption. As such, patients are at increased risk for osteopenia and fractures [1,20]. Bone marrow crowding and low levels of normal mature immunoglobulins in MM result in the suppression of immune cells, resulting in infection as a common complication [1]. High levels of serum paraproteins can also increase blood viscosity, increasing the risk of dyspnea, transient ischemic attack, retinal hemorrhage, blurry vision, seizure, and deep venous thrombosis. The consequent reduction in platelet count can also lead to easy bruising and bleeding [2,3].

## 5. Current Treatment of Multiple Myeloma

### 5.1. Overview

Treatment modalities have improved concomitantly with the progression in understanding of the molecular pathogenesis of MM over the past twenty years [21]. The use of corticosteroids (prednisone and dexamethasone) and alkylating agents (mainly melphalan and cyclophosphamide) as standard therapies began in the mid-1960′s. Since the 1990′s, treatment protocols have included autologous stem cell transplant (ASCT) for eligible patients [21]. Drug classes, including immunomodulatory drugs (IMiDs), proteasome inhibitors (PIs), and monoclonal antibodies (mAbs), have become the cornerstone of modern multiple myeloma therapy [21,22]. Combinations of these drug classes have become a standard of care in newly diagnosed transplant-eligible or -ineligible patients, and are utilized in triplet or quadruplet regimens relative to each patient’s unique clinical profile [23].

Despite these advances, a definitive cure for this disease remains elusive; relapse is inevitable, and refractory disease requiring salvage therapy remains a considerable challenge [22,24,25,26]. This has prompted the development of new biologic agents and immunotherapy in the past decade [21,26,27,28]. The optimal sequence and combination of novel immunotherapeutic strategies remains to be determined. Current treatment options continue to evolve as we increase our understanding of multiple myeloma’s complex molecular pathophysiology and resulting clinical implications (Table 1).

### 5.2. Anti-BCMA Compounds Under Development

In 2015, the FDA approved two mAbs, daratumumab and elotuzumab, which selectively target MM cell glycoproteins CD38 and SLAMF7, respectively [21,69]. Several novel immunotherapeutic approaches have emerged since this time. New therapies can target plasma cell-specific antigens to offer an innovative approach to treatment optimization and options for relapsed/refractory disease [21,22]. B-cell maturation antigen (BCMA), a soluble transmembrane glycoprotein overexpressed in MM cells, represents an important target for novel therapeutics. These modalities include antibody-drug conjugates (ADCs) (belantamab mafodotin), bispecific T-cell engagers (BiTEs) (AMG 420), and CAR T-Cell Therapies [21,70].

#### 5.2.1. Anti-BCMA Antibody-Drug Conjugates (ADCs)

Antibody-drug conjugates act as a carrier to deliver cytotoxic agents into MM cells, leading to targeted tumor cell lysis with reduced toxicity in non-targeted tissues. They are composed of a mAb, a linker connecting the drug to the antibody, and the cytoxic drug. Similar to belamaf, a first-in-class anti-BCMA ADC, there are several emerging anti-BCMA therapeutics currently under development for use in multiple myeloma.

AMG 224 is a compound consisting of an anti-BCMA IgG1 antibody conjugated via a linker (4-[N-maleimidomethyl] cyclohexane-1-carboxylate) to mertansine, an anti-tubulin inhibitor [71]. There is an ongoing phase I study of AMG 224 as monotherapy in heavily pre-treated patients with relapsed or refractory multiple myeloma (NCT02561962). Similarly, MEDI2228 is an ADC using tesirine, a pyrrolobenzodiazepine dimer, as a toxic payload to MM cells. Tesirine is a DNA cross-linking agent with site-specific conjugation to BCMA-Ab1 via a valine-alanine dipeptide linker. MEDI2228 is internalized and trafficked to the lysosome where tesirine is released, resulting in DNA damage, myeloma cell and myeloma progenitor cell death [72]. Current clinical testing is ongoing for its use in the treatment of RRMM (NCT03489525).

HDP-101 is a compound in a new class of ADCs called antibody-targeted amanitin conjugates (ATAC). Amanitin, a toxin contained in the *Amanita phalloides* death cap mushroom, selectively binds to and inhibits the RNA polymerase II subunit A with high affinity. This results in a >1000-fold decrease in transcription and protein synthesis, leading to cell apoptosis and death. Of note, HDP-101 utilizes chemically synthesized amanitin as its toxic payload conjugated to the anti-BCMA mAb via a cathepsin B protease linker [73,74]. A Phase Ia/Ib dose escalation and expansion study is expected to begin in early 2021 to evaluate the effect of HDP-101 in patients with RRMM.

Other anti-BCMA ADCs currently in phase I trials for patients with RRMM include CC-99712 (NCT04036461) and SEA-BCMA, a naked anti-BCMA mAb without conjugate (NCT03582033).

#### 5.2.2. Anti-BCMA Bi-Specific Antibodies (BiAbs)

Bi-specific antibodies are a novel potential therapy for patients with multiple myeloma. Bi-specific T-cell engaging antibody (BiTEs) are a specific type of BiAb that transiently connect immune and tumor cells through their interaction with both CD3 on the T-cell and tumor antigens on the surface of target tumor cells. The molecules are designed with two domains, one that binds CD3ε in the T-cell receptor (TCR) complex and the other that recognizes BCMA on MM cells. The binding of CD3ε leads to activation of cytotoxic T-cells which release perforin and granzymes to lyse the targeted tumor cells, and interferon-γ which activates macrophages and immune cells [75,76].

Several anti-BCMA BiAbs are in ongoing clinical trials for patients with MM. These include AMG 420 (NCT03836053), AMG 701 (NCT03287908), CC-93269 (NCT03486067), Teclistamab (NCT04557098, NCT03145181, NCT04586426, NCT04108195), TNB-383B (NCT03933735), PF-06863135 (NCT03269136, NCT04649359), REGN5458 (NCT03761108) and REGN5459 (NCT04083534).

### 5.3. Novel Agents

Novel agents have further expanded the available therapeutic options for patients with relapsed/refractory MM. Selinexor is a first-in-class Exportin-1 (XPO-1) inhibitor, granted accelerated approval in July 2019 for patients with penta-refractory multiple myeloma. Other therapeutics in this class include Filanesib (ARRY-520) and Venetoclax (ABT-199), a kinesin spindle protein inhibitor (KSP) and selective BCL-2 inhibitor, respectively. Filanesib is the only KSP inhibitor that has shown anti-tumor activity in clinical trials. It has demonstrated clinical efficacy in heavily pretreated multiple myeloma patients and may be useful in combination with standard MM backbones, such as PIs and IMiDs [61,62]. Both Filanesib and Venetoclax are under clinical investigation for extended indications, alone and in combination regimens in patients with multiple myeloma.

Immunotherapy via adoptive cell transfer (ACT) is also a promising investigational MM treatment. Current trials are exploring the use of autologous chimeric antigen receptor (CAR)-transduced T-cells, in which host T-cells are engineered with viral vector recombinant DNA techniques. CAR T-cells are then used to initiate a targeted immune response against antigens specific to MM cells [21,26,77].

### 5.4. Hematopoietic Stem Cell Transplant Eligibility

Given the recent expansion in therapeutic options, individualized treatment for MM is ever evolving and guided by a variety of clinical parameters [24,27]. At this time, eligibility for ASCT and risk-stratification are predominant determinants of the treatment course of newly diagnosed MM [15,23]. ASCT remains the standard for first-line treatment of newly diagnosed MM; therefore, phases of management are generally defined, relative to transplant eligibility [24,78]. Various applications of the aforementioned treatments are utilized, depending on a patient’s transplant status. Transplant eligibility is primarily determined by age and existing comorbidities, since these factors predispose patients to toxicity and influence a patient’s ability to endure treatment [24,79]. The majority of randomized trials limit ASCT to patients ≤ 65 years of age without significant comorbidities, although consensus regarding an age cutoff has not been established, and practice varies across institutions [79]. Contraindications for ASCT include significant cardiac or pulmonary disorders [24]. Transplant-eligible patients typically undergo 3–4 cycles of the current standard induction therapy, which is a triplet regimen consisting of bortezomib, lenalidomide, and dexamethasone (VRd). Transplant-ineligible patients undergo 8–12 cycles of VRd induction therapy [23,79,80]. Patients undergoing ASCT are treated with cytokines or chemotherapy, after which hematopoietic stem cells are mobilized into peripheral blood and harvested by apheresis [81]. The stem cells can then be used for marrow reconstitution following high-dose chemotherapy. The standard maintenance therapy for both transplant ineligible patients and eligible patients following ASCT is lenalidomide [15].

## 6. Belantamab Mafodotin Drug Info

In August 2020, the FDA granted accelerated approval to belantamab mafodotin-blmf (belamaf) as a monotherapy treatment for relapsed or treatment-refractory multiple myeloma. Belamaf is a first-in-class biologic for patients who have previously attempted four other treatments, including an anti-CD38 monoclonal antibody, a proteasome inhibitor, and an immunomodulatory agent [82,83]. As the first approved anti-B cell maturation antigen (BCMA), the use of belamaf may have a large impact on improving progression-free survival in patients with multiple myeloma who have limited remaining treatment options. BCMA is a cell surface receptor required for the survival of plasma cells. The expression of BCMA can be detected on all CD138+ myeloma cells, but it is not expressed in any other tissues. This receptor specificity allows belamaf to target only the malignant MM plasma cells [7,84].

Belantamab mafodotin is associated with a high incidence (≥20%) of keratopathy [85]. To mitigate such risks, an ophthalmic exam is recommended prior to and during belamaf therapy in order to assess baseline vision and possible adverse eye effects. Dosage can be reduced or held if ocular toxicity such as blurry vision, dry eyes, or corneal ulcers occurs. Belamaf should be discontinued if ocular toxicity is severe [82]. Other less common adverse effects, such as thrombocytopenia, infusion-related reactions, pyrexia, fatigue, nausea, constipation, diarrhea, arthralgia, back pain, decreased appetite, and upper respiratory infection, have been reported [86]. The most common grade 3 or 4 laboratory abnormalities (≥5%) include decreases in neutrophils, lymphocytes, platelets, and hemoglobin, along with increases in gamma-glutamyl transferase and creatinine [82].

There is a paucity of data on the use of belantamab during pregnancy and breastfeeding. Because belantamab is a large protein molecule, the amount excreted in breastmilk is postulated to be very low. However, belantamab is conjugated with mafodotin, a small-molecule toxin, which may be excreted into milk. As such, it is recommended that patients use effective contraception and avoid breastfeeding while taking the medication and for 3 months after the last dose [87].

## 7. Belantamab Mafodotin Mechanism of Action

### 7.1. Antibody Drug Conjugate (ADC)

Belantamab mafodotin (Blenrep, GSK2857916 or J6M0-MMAF) is an antibody-drug conjugate (ADC) that demonstrates a multifaceted mechanism of action based on three main components. ADCs are a new class of cancer therapeutics that confer unique pharmacologic activity via mAbs covalently conjugated to a cytotoxic agent via a specialized linker [28]. The mAb component of an ADC selectively targets tumor cells and elicits a host immune response, while simultaneously delivering a cytotoxic payload to the cell [25,86]. Belamaf consists of a humanized, afucosylated IgG1 mAb conjugated to monomethyl auristatin-F (MMAF) via a protease-resistant maleimidocaproyl linker [25,70].

### 7.2. Target Antigen-B-Cell Maturations Antigen (BCMA)

The high specificity of belamaf for MM cells is a hallmark feature derived from the mAb component, which targets B-Cell Maturation Antigen (BCMA). BCMA, a member of the tumor necrosis factor receptor superfamily, is a notable tumor-associated antigen of particular interest due to almost exclusive BCMA expression on mature B-cells and plasma cells. BCMA is integral to plasma cell maturation and differentiation. BCMA is also overexpressed during the malignant transformation of plasma cells, making it an ideal pharmacologic target in the treatment of MM [21,25,70]. B-cell activating factor (BAFF) and APRIL (a proliferation-inducing ligand) are high-affinity ligands for BCMA that promote proliferation and viability of MM cells in the bone marrow. BAFF is a BCMA agonist that induces differentiation, proliferation, and antibody production [21,70]. The binding of belantamab to BCMA receptors impedes the pro-survival cytokine-signaling effects of BAFF and APRIL on malignant plasma cells [21,28].

### 7.3. Afucosylated Monoclonal IgG1 Antibody

Belamaf induces enhanced tumor cell lysis via natural killer cell-mediated antibody-dependent cell-mediated cytotoxicity (ADCC) and antibody-dependent cellular phagocytosis (ADCP) [28,70,86]. While naturally occurring IgG antibodies exhibit significant core-fucosylation on the N-glycan of the Fc region, the IgG1 mAb in belantamab mafodotin is afucosylated. The removal of these fucosyl groups enhances IgG1 Fc binding affinity to FcγRIIIa (CD16) on natural killer cells, which is a well-known strategy for augmenting effector cell ADCC of cancer cells [21,25,88].

### 7.4. Monomethyl Auristatin-F (MMAF) and Linker

After belamaf binds, the mAb drug complex is internalized, allowing MMAF to induce apoptosis [82]. MMAF inhibits tubulin polymerization to disrupt microtubules and arrest myeloma cells at the G2/M checkpoint [21]. Of note, the protease-resistant properties of the linker used in belamaf requires lysosomal degradation of the attached antibody for the release of MMAF in the cell. The use of non-cleavable linkers helps to prevent the side effects of premature release of the toxic payload before it is internalized by the MM cells [25].

## 8. Pharmacokinetics

The pharmacokinetic profile of belantamab mafodotin was evaluated as a secondary endpoint of a dose-escalation and expansion of the phase I trial. Methods, including population pharmacokinetics and conventional allometry demonstrated linear, dose-proportionality. Current dosage and administration recommendations are based on Phase II DREAMM-2 study findings, which supported recent accelerated FDA approval of belamaf.

Clinically significant demographic differences in pharmacokinetics of belamaf were not observed in the study patients (34–89 years, male vs. female, white vs. black, 42–130 kg in weight). Additionally, inconsistencies in pharmacokinetics were not observed in physiologic disturbances, including mild hepatic impairment (defined as total bilirubin ≤ULN and AST >ULN or total bilirubin 1 to ≤1.5 x ULN and any AST) and mild renal impairment (defined as eGFR 30–89 mL/min/1.73 m^2^). Further investigation is needed to determine the implications of severe renal impairment (ESRD with eGFR <15 mL/min/1.73 m^2^) or moderate to severe hepatic impairment on the pharmacokinetics of belamaf, as these data are not available at this time [82].

### 8.1. Absorption and Distribution

Belantamab mafodotin is administered as an intravenous infusion, and FDA guidelines advise a dosage of 2.5 mg/kg over the course of 30 min every three weeks [82]. The 2.5 mg/kg dose was further investigated, based on its preferable safety profile and comparable anti-myeloma activity to the 3.4 mg/kg dose [80]. An exposure–response relationship was not observed at 2.5 or 3.4 kg/mg after controlling for the effect of existing disease-related variables [89]. There was no reported evidence of large QTc prolongation (>10 ms) at the recommended dosage of 2.5 mg/kg once every three weeks.

Levels of belantamab, parent antibody, and total antibody were observed at maximum concentrations at the completion of the 30-min infusions, in comparison to the activated cytotoxic drug (cys-mcMMAF) levels, which were shown to peak about 24 h after doses were administered [89]. Approximately 70% of accumulation was observed with the above dosing regimen. The time to reach a steady state was approximately 70 days. The mean steady-state volume of distribution was 11 L (15%) [82]. The steady-state volume of distribution (V_ss_) was documented at 4.2 L, which aligns with the characteristic V_ss_ for a monoclonal antibody. This V_ss_ value suggests that the distribution of belamaf is mainly restricted to the systemic circulation and interstitial space [89].

### 8.2. Metabolism

The mechanism of metabolism is anticipated to occur via the catabolic processing of the monoclonal antibody portion into single amino acids and small peptides. The cyclic isomer form of the active cytotoxic payload, cys-mcMMAF, was metabolized by hydrolysis and dehydration in vitro [82].

### 8.3. Elimination

The terminal phase half-life was 12 days following the first dose and 14 days when a steady state was reached [82]. Slow total plasma clearance was noted at 0.37 L/d, with a gradual decrease in clearance over time. Compared with plasma clearance after the first dose (0.9 L/day (42%)), the total plasma clearance was roughly 22% lower at steady state [82].

## 9. Clinical Studies: Safety and Efficacy

### 9.1. Phase I Studies: DREAMM-1 Trial

DREAMM-1, a phase I clinical trial, analyzed the safety and clinical efficacy of GSK2857916 (belantamab mafodotin) and consisted of two parts. Part I, the dose-escalation phase, explored the drug’s safety, tolerability, maximum tolerated dose (MTD), and recommended phase II dose (RP2D) in multiple myeloma patients with progressive and refractory disease (Table 2). A total of 38 patients were treated in part 1 (0.03–4.60mg/kg) via one-hour intravenous infusions once every three weeks for a maximum of 16 total treatments. The National Cancer Institute Common Criteria for Adverse Events version 4.0 was utilized to define adverse events (AEs). In part I, 53% (20/38) of patients developed corneal AEs, 90% of which were categorized as grade I–II. Grade IV–V corneal AEs did not occur [89]. Of note, all patients were pretreated with steroid eye drops prior to belamaf infusion due to evidence of monomethyl auristatin F (MMAF)-induced corneal toxicity occurring with previously studied antibody-conjugate drugs [90]. The most common grade III AE was anemia, developing in 16% of patients. Thrombocytopenia accounted for 4 of the 7 grade IV AEs. Overall, belamaf was well tolerated with no MTD or dose-limiting toxicities developing in part I of the study. An RP2D of 3.40 mg/kg was determined based on a 100% response rate occurring in three patients at this dosage with additional consideration to lower tolerability observed at higher dosages [89].

The primary endpoint of part II, the expansion phase, focused on the tolerability, safety, PK, and clinical activity of the RP2D (3.4 mg/kg) (Table 2). The most frequently observed AEs in these patients was blurry vision (45%) at a grade 1–2 severity. The data are in concordance with a previous study concerning ocular AE in antibody-combination therapy in which blurred vision was the most commonly observed sequela [90]. Thrombocytopenia was, once again, the most common grade IV AE, occurring in approximately 9% of patients. As in part I, no grade V AEs were observed. Additionally, no deaths were attributed to the drug throughout the phase I trial. Concerning the efficacy of the RP2D, a 60% overall response rate (ORR) was achieved with a 95% CI: (42.1–76.1). A stringent complete response (sCR) occurred in 3% of patients (*n* = 1), a complete response (CR) was observed in 6% of patients (*n* = 2), and a very good partial response (VGPR) occurred in >40% of patients (*n* = 16). Of those previously treated with greater than five lines of therapy (29 patients), ORR was 46.2% (95% CI: 19.2–74.9). Approximately 60% (*n* = 38) of patients, refractory to proteasome inhibitor therapy, achieved an ORR of 58.8% (95% CI: 40.7–75.4). At this stage in the study, the median progression-free survival (PFS) was approximated at 7.9 months (95% CI: 3.1–not estimable) [89].

An update in the phase I trial was published after an additional 14 months of follow-up, revealing an identical ORR, but with one additional response occurring in both the sCR (two patients) and CR (three patients) categories (Table 2). Median PFS was approximated at 14.3 months (95% CI: 3.1–not estimable), with the average first response occurring within two months of treatment. Concerning those without a prior history of daratumumab therapy (21 patients), an ORR was achieved in 71.4% (95% CI: 47.8–88.7). Of patients refractory to previous daratumumab therapy (13 patients), 38.5% attained an ORR (95% CI: 13.9–68.4). This data, although from a small cohort, may suggest much lower drug activity in daratumumab refractory patient populations. Safety profiles reflected previous trends with corneal events as the most commonly experienced sequela of treatment [7]. In summary, this phase I clinical trial achieved its primary endpoint, as the RP2D proved efficacious in the treatment of a small cohort of refractory multiple myeloma patients with minimization of serious AE.

### 9.2. Phase II Studies: DREAMM-2 Trial

DREAMM-2, a randomized two-arm phase II study, focused on the safety and efficacy of belamaf in patients with refractory multiple myeloma who were allocated into two dosage cohorts: 2.5 mg/kg (97 patients) or 3.4 mg/kg (99 patients) (Table 2). These dosages were chosen based on the positive clinical responses achieved in the phase I study. The primary endpoint entailed the ORR achieved in both cohorts of treatment. Of those who received 2.5 mg/kg of treatment, an ORR of 31% (95% CI: 20.8–42.6) was observed, with 18 patients (18.5%) achieving a VGPR (median time to follow-up 6.3 months). Of those who received 3.4 mg/kg of therapy, a 34% ORR (95% CI: 23.9–46) was achieved (median time to follow-up 6.9 months). The median time to response was within two months for both cohorts with an accompanying progression-free survival of 2.9 months and 4.9 months in the lowest and highest dose groups, respectively.

As with the DREAMM-1 trial, corneal events, thrombocytopenia, and anemia were the most prevalent AEs reported. Specifically, corneal events occurred in 70.5% of the 2.5 mg/kg group and in 76.8% of the 3.4 mg/kg group, with 27% and 33% of patients experiencing grade III–IV AEs, respectively. Interestingly, topical steroid prophylaxis was not proven beneficial in preventing ocular AEs [66]. Twenty percent of patients in the low dose arm experienced grade III–IV thrombocytopenia in comparison to 33% of patients who received the higher dose therapy. Over 40% of patients in both cohorts had evidence of serious AEs, with one death occurring in each cohort that was not unattributable to the drug (sepsis in 2.5 mg/kg group; hemophagocytic lymphohistiocytosis in 3.4 mg/kg group). Of note, patients included in DREAMM-2 had more progressive disease and were more refractory to previous medical therapy (median of 6 prior lines of treatment in 2.5 mg/kg cohort; 7 lines in 3.4 mg/kg cohort) in comparison to those included in the DREAAM-1 trial [65,89].

Due to the known corneal toxicity potential of MMAF, corneal AEs were further analyzed, utilizing data from the 2.5 mg/kg cohort in the DREAMM-2 trial (Table 2). As in the DREAMM-1 trial, patients received prophylactic steroid eye solution before belamaf therapy. Prior studies concerning antibody-drug conjugate associated epithelial changes have shown inconsistent evidence of steroid prophylaxis efficacy; however, studies focused specifically on the prevention of microcyst-like epithelial changes (MEC) are scarce. MEC were defined as the disruption of epithelium architecture noted by ophthalmologist-conducted slit-lamp examination. These lesions manifested in patients as small polymorphous opacities limited to the corneal epithelium. Such changes are theorized to result from drug-induced apoptosis, following macropinocytosis of belamaf’s MMAF moiety by corneocytes. Patients also received a best-corrected visual acuity (BCVA) assessment prior to participation in the trial. The MEC and BCVA were subsequently graded utilizing the keratopathy and visual acuity (KVA) scale. According to data analysis, grade I–II (mild-moderate) MEC occurred in 25% of patients, grade III events were observed in 45%, with only 1% of patients experienced grade IV MEC (severe) [66].

The most common ocular symptoms included blurred vision (21% of patients) and dry eyes (15% of patients). Of those who developed any ocular side-effects, 25% had evidence of MEC following the first administration of belamaf. Despite MEC occurring in the majority of patients, nearly 50% of patients with grade II or higher MEC recovered to baseline corneal architecture. Although these results are promising, the use of topical steroids potentially confounds the accuracy of this data. Of those who reported symptoms of blurry vision and dry eyes, 63% and 79% achieved recovery, respectively. As previously mentioned, steroid prophylaxis did not appear to have a statistically significant beneficial impact on the prevention of corneal events in the DREAMM-2 trial. Change in BCVA occurred in 54% of patients, with the majority of events labeled as grade III. Of those with grade II or greater change, approximately 60% returned to baseline BCVA as in the last follow-up [66]. Whilst the DREAMM-2 trial provides insight into the ocular effects of belamaf, additional research regarding incidence and resolution of MEC is necessary to determine the best ophthalmologic management of these patients with concurrent optimization of therapy. Current clinical trials are planned to further characterize corneal epitheliopathy in patients treated with belamaf (NCT04549363).

### 9.3. Case Series

A case series including five patients treated with 3.4 mg/kg belamaf once every 3 weeks aimed to characterize belamaf-related corneal events. Topical steroid prophylaxis was utilized prior to belamaf administration, and symptoms of blurry vision and dry eyes were managed with preservative-free (PF) eye lubricant drops and increased frequency of steroid use. All five patients experienced corneal events, with three of the five developing grade III AEs over a median follow-up period of 32.6 months. Due to these ocular sequelae, each patient required dose interruptions, and all patients received two dose reductions throughout the duration of treatment. No patients experienced grade IV or V corneal AEs. Of note, upon the cessation of belamaf therapy, all patients attained resolution of grade III or above AEs within one year of the last dose. Although the optimal management of belamaf-related ocular events is yet to be determined, the authors of this study recommend limiting the extended use of topical steroids while utilizing PF lubricant drops and dose modifications. Regarding belamaf efficacy in this small study, two patients attained an sCR, with a CR occurring in one patient, and a VGPR achieved in two patients [91].

### 9.4. Indirect Comparison Study

One study analyzed the clinical efficacy of belamaf monotherapy versus selinexor (nuclear export protein 1 inhibitor) plus dexamethasone (sel+dex) via an indirect comparison in multiple myeloma patients refractory to anti-CD38 therapy. Data from the DREAM-2 trials were utilized to generate matching-adjusted indirect comparisons. Belamaf treatment was found to be superior to the sel+dex duration of response (Hazard Ratio: 0.34) and overall survival (Hazard Ratio: 0.60). No statistical significance was observed in the overall response rate, progression-free survival, or time to response between the two treatment regiments [92]. Direct comparisons with increased statistical power will be required to provide convincing evidence of belamaf superiority with regard to sel+dex in the treatment of refractory multiple myeloma.

### 9.5. Future Studies

Following the results of the DREAMM-1 and two trials, multiple studies concerning belantamab mafodotin are currently in the pipelines, with several of these studies actively recruiting patients (Table 3). The focuses of these upcoming trials include the efficacy and safety of belamaf in comparison to pomalidomide plus low-dose dexamethasone (DREAMM-3), belamaf in combination with pembrolizumab (DREAMM-4), and belamaf in combination with lenalidomide or bortezomib plus dexamethasone (DREAMM-6) [93,94,95]. The DREAMM-5 trial aims to analyze the efficacy of belamaf plus various anti-cancer drugs, including GSK335609 (inducible T-cell costimulatory agonist), nirogacestat (gamma secretase inhibitor), and GSK317498 (selective OX40 agonist) [96,97,98]. At the time of this review, no direct head-to-head comparisons of belamaf vs. other agents have been performed; however, the DREAMM-7 trial plans to study the clinical efficacy of belamaf in combination with bortezomib plus dexamethasone versus daratumumab in combination with bortezomib plus dexamethasone. DREAMM-8 plans to compare the safety and efficacy of belamaf in combination with pomalidomide and dexamethasone versus bortezomib in combination with pomalidomide and dexamethasone. The DREAMM-9 and 10 trials plan to compare belamaf versus the standard of care for multiple myeloma [98]. DREAMM-12 and 13 trials will assess safety and tolerability of belamaf in patients with normal or impaired renal or hepatic function, respectively, So far, belamaf has provided promising evidence of clinical efficacy with manageable adverse effects for patients with progressive and refractory multiple myeloma; however, future DREAMM trials are required to determine an optimal treatment approach and effective risk-management strategies.

## 10. Conclusions

Multiple myeloma is a hematologic malignancy characterized by the excessive clonal proliferation of plasma cells. The treatment of multiple myeloma presents a variety of unique challenges due to the complex molecular pathophysiology and incurable status of the disease at this time. Given that MM is the second most common blood cancer with a characteristic and unavoidable relapse/refractory state during the course of the disease, the development of new therapeutic modalities remains crucial.

Belantamab mafodotin (belamaf) was designated as a breakthrough therapy for heavily pretreated patients with relapsed/refractory multiple myeloma in November 2017 and continues to show promise as a unique modality for future treatment regimens with recent accelerated FDA approval. Belamaf offers a unique combination of therapeutic mechanisms as an antibody drug conjugate with exclusive antitumor activity in MM tumor cells via BCMA binding. The IgG1 mAb component not only confers specificity but also induces an immune response, resulting in ADCC and ADCP along with the induction of apoptosis via the toxic payload (MMAF).

While several investigational therapeutics with anti-BCMA activity are being investigated at this time, belantamab mafodotin offers the benefits of an off-the-shelf therapy that is administered once every three weeks. The most prominent adverse events include corneal toxicity, thrombocytopenia, and anemia associated with the MMAF payload. Specific prophylaxis or treatment guidelines for belamaf-related keratopathy are not known, as the underlying mechanism of this toxicity remains unclear. Corticosteroid eye drops were not found to reduce the frequency of keratopathy in clinical studies. Current management strategies include mainly dose modifications (delays and reductions) to allow for the regeneration of corneal epithelial cells and PF lubricant drops. Other strategies include cooling eye masks or vasoconstrictors administered at the start of the infusion. However, the true benefit of these mitigation strategies remains unclear.

Based on the DREAMM-2 evidence of 31% of patients achieving an ORR and a very good response rate observed in 18.5% of individuals, belamaf is a valid treatment option for MM patients who have exhausted multiple standard therapies. Future phase III studies are necessary to evaluate efficacy in comparison to other standard treatment modalities. Additional planned DREAMM trials will be aimed at determining the utility of belantamab mafodotin in combination with other current MM drugs, including protease inhibitors, monoclonal antibodies, and corticosteroids. Further research is needed to define ocular toxicity mitigation strategies, the potential benefits of belamaf use for early MM, potential synergistic therapeutic combinations, and the durability of drug-induced immunogenic responses to myeloma cells. 

## Figures and Tables

**Table 1:** Current Therapeutic Considerations for Multiple Myeloma.

Strategy	Name of the Drug	Mechanism of Action	References
Corticosteroids	Prednisone Dexamethasone	Anti-inflammatory and anti-proliferative effects on myeloma cells	[29]
Conventional Chemotherapy	Cyclophosphamide Doxorubicin Melphalan Bendamustine	Alkylating agent Inhibits topoisomerase II; Intercalates into DNA Alkylating agent Alkylating agent	[30] [31] [32,33] [34,35]
Immunomodulatory Drugs (IMiDs)	Thalidomide Lenalidomide Pomalidomide Avadomide (CC-122) * Iberdomide (CC-220) *	All: Inhibit production of TNF-a, IL-6, IL-8, VEGF; activate caspase-8 IL-6 inhibition; caspase-8 activation Inhibits Akt phosphorylation; co-stimulates CD28 Co-stimulates CD28 Cereblon E3 ligase modulator Cereblon E3 ligase modulator	[36] [36] [37,38] [39] [39]
Proteasome Inhibitors (PIs)	Bortezomib Carfilzomib Ixazomib Oprozomib * Marizomib * Delanzomib *	Reversibly binds to CT-L/LMP7 subunit; binds C-L /LMP2 and T-L subunits with lower affinity Irreversibly binds to CT-L/LMP2 subunit; binds C-L/LMP2 and T-L subunits at high doses Binds to beta 5 subunit of 20s proteasome Irreversibly binds to CT-L /LMP7 subunit Binds CT-L/LMP7 and T-L subunits with high affinity; binds C-L/LMP2 subunit with lower affinity Reversibly inhibits CT-L/LMP7 and C-L /LMP2 subunits	[40] [41] [42] [43] [43] [43]
Histone Deacetylase (HDAC) Inhibitors	Panobinostat Romidepsin Ricolinostat Citarinostat *	Pan-Histone Deacetylase Inhibitor Histone Deacetylase Inhibitor Histone Deacetylase 6 Inhibitor Histone Deacetylase 6 Inhibitor	[44] [45] [46,47] [48]
Monoclonal Antibodies (mABs)	Daratumumab Elotuzumab Denosumab Siltuximab Felzartamab (MOR202) * Isatuximab TAK-079 *	Anti-CD38 Anti CS1/SLAMF7 Anti-RANKL Anti-IL6 Anti-CD38 Anti-CD38 Anti-CD38	[49] [50,51] [52] [53] [53] [54] [55]
Immunotherapies	Durvalumab Pembrolizumab Nivolumab Nelfinavir BiTE * CAR-T *	Anti-PDL1 Anti-PD1 Anti-PDL1 Protease Inhibitor Anti-BCMA Anti-BCMA	[56,57] [56,57] [56,57] [57] [58,59] [60]
Novel Agents	Filanesib * Venetoclax * Selinexor	Kinesin Spindle Protein (EG5/KIF11) Inhibitor Selective Inhibitor of BCL-2 Inhibitor of XPO1-mediated nuclear export protein	[61,62] [63] [64]
Antibody-Drug Conjugates (ADCs)	Belantamab mafodotin Lorvotuzumab mertansine * Milatuzumab doxorubicin * Indatuximab ravtansine *	Anti-BCMA Anti-CD56 Anti-CD74 Anti-CD138	[65,66] [67] [68] [68]

* currently under clinical development for use in multiple myeloma.

**Table 2:** Clinical Efficacy and Safety.

Author (Year)	Groups Studied and Intervention	Results and Findings	Conclusions
Trudel S. et al. (2018) [7,89]	DREAMM-1 trial: Patients with progressive and refractory multiple myeloma were treated with belamaf once every 3 weeks for a maximum of 16 treatments. Thirty-eight patients were included in the dose-escalation phase, with the dose-expansion phase consisting of thirty-five patients.	Dose escalations phase: Three patients receiving 3.4 mg/kg of drug therapy achieved an 100% response rate. Corneal events were the most commonly experienced adverse effect (AE) occurring in 53% of patients. Anemia was the most common grade III AE, and thrombocytopenia was the most frequently observed grade IV AE. A dosage of 3.4 mg/kg was designated as the recommended phase II dose (RP2D) based on data suggesting an acceptable efficacy and safety profile. Dose expansion phase: A 60% overall response rate (ORR) was achieved in those receiving 3.4 mg/kg of therapy with >40% of patients achieving a very good response rate (VGPR). Stringent complete response (sCR) was attained in one patient.	Data from this phase I study suggest belamaf is efficacious and well-tolerated in those with multiple myeloma refractory to multiple lines of therapy.
Lonial S. et al. (2020) [65,66]	DREAMM-2 trial: Analyzed the clinical efficacy and safety of belamaf in two treatment cohorts at dosages 2.5 mg/kg (97 patients) and 3.4 mg/kg (99 patients). Both groups had received a median of 6 prior lines of therapy.	2.5 mg/kg cohort: 31% of patients achieved an ORR with a VGPR observed in 18.5% of individuals. An estimated progression free survival (PFS) of 2.9 months was observed. 3.4 mg/kg cohort: An ORR was attained in 34% of patients in this treatment group. PFS was approximately 4.9 months. Median time to response was within 2 months in both treatment cohorts. Corneal events, anemia, and thrombocytopenia were the most commonly occurring AE with evidence of serious AEs observed in over 40% of patients at both dosages. Nearly half of patients with grade II ocular AEs had return to baseline corneal architecture following cessation of belamaf therapy.	Belamaf at dosages 2.5 mg/kg and 3.4 mg/kg proved to be clinically efficacious in patients that were refractory to a median of ≥6 lines of therapy. The 2.5 mg/kg cohort experienced similar results with reduced AE in comparison to those receiving higher dosage. Phase III studies are needed to evaluate efficacy in comparison to standard therapy modalities.

**Table 3:** Ongoing Clinical Trials of Belantamab Mafodotin in Multiple Myeloma.

Phase	Identifier	*EN*, *n*	Drug(s)	Indication	Prior Anti-myeloma Treatments, *N* of lines	Status
Phase III	NCT04162210, DREAMM-3	380	Arm A: Belantamab mafodotin Arm B: Pomalidomide + Dexamethasone	RRMM	≥2	Recruiting
Phase I/II	NCT03848845, DREAMM-4	40	Belantamab mafodotin Pembrolizumab	RRMM	≥3	Active, Not Recruiting
Phase I/II	NCT04126200, DREAMM-5	464	Belantamab mafodotin GSK3174998 GSK3359609 Nirogacestat Dostarlimab	RRMM	≥3	Recruiting
Phase I/II	NCT03544281, DREAMM-6	152	Arm A: Belantamab mafodotin, Dexamethasone, Lenalidomide Arm B: Belantamab mafodotin, Dexamethasone, Bortezomib	RRMM	≥1	Recruiting
Phase III	NCT04246047, DREAMM-7	478	Arm A: Belantamab mafodotin, Bortezomib, Dexamethasone Arm B: Daratumumab, Bortezomib, Dexamethasone	RRMM	≥1	Recruiting
Phase III	NCT04484623, DREAMM-8	450	Arm A: Belantamab mafodotin, pomalidomide, dexamethasone Arm B: Bortezomib, pomalidomide, dexamethasone	RRMM	≥1	Recruiting
Phase I	NCT04091126, DREAMM-9	144	Belantamab mafodotin Bortezomib Lenalidomid Dexamethasone	NDMM TI	-	Recruiting
Phase I	NCT04398745, DREAMM-12	36	Belantamab mafodotin	RRMM	≥2	Recruiting
Phase I	NCT04398680, DREAMM-13	24	Belantamab mafodotin	RRMM	≥2	Not Yet Recruiting
Phase III	NCT04549363	25	Belantamab mafodotin	RRMM, prior or current treatment with belamaf	-	Not Yet Recruiting
Phase I/II	NCT03715478, ALGONQUIN	62	Belantamab mafodotin Pomalidomide Dexamethasone	RRMM	≥2	Recruiting
Phase I	NCT04177823	5	Belantamab mafodotin	RRMM	≥2	Active, Not Recruiting
Phase II	NCT04680468	47	Belantamab mafodotin prior to melphalan + ASCT	RRMM	≤2	Not Yet Recruiting
Phase II	NCT03525678	221	Belantamab mafodotin (frozen liquid) Belantamab mafodotin (lyophilized powder)	RRMM	≥3	Active, Not Recruiting
Phase I	NCT03828292	14	Part 1: Belantamab mafodotin monotherapy Arm A: Belantamab mafodotin + Bortezomib/Dexamethasone Arm B: Belantamab mafodotin + Pomalidomide/Dexamethasone	RRMM	≥2	Recruiting
